# Exposure to Advertisements and Marijuana Use Among US Adolescents

**DOI:** 10.5888/pcd14.170253

**Published:** 2017-11-30

**Authors:** Hongying Dai

**Affiliations:** 1 Health Services & Outcomes Research, Children’s Mercy Hospital, Kansas City, Missouri; 2 University of Missouri–Kansas City, Kansas City, Missouri

## Abstract

**Introduction:**

This study examined whether exposure to marijuana advertisements was associated with current marijuana use and frequency of use among US adolescents in grades 8, 10, and 12.

**Methods:**

Weighted estimates of exposure to marijuana advertisements and marijuana use from the 2014 and 2015 Monitoring the Future studies were investigated. Factors associated with the prevalence and frequency of marijuana use were analyzed by using logistic regression and linear regression models, respectively.

**Results:**

Of all respondents (n = 12,988), 13.8% reported marijuana use in the past 30 days. Exposure to marijuana advertisements was prevalent among adolescents, with 52.8% reporting exposure from internet advertisements, 32.1% from television advertisements, 24.1% from magazine or newspaper advertisements, 19.7% from radio advertisements, 19.0% from advertisements on storefronts, and 16.6% from billboards. In the multivariable analysis, current use of marijuana among adolescents was associated with exposure to marijuana advertisements on storefronts (adjusted odds ratio [OR] = 1.4, *P* < .001), magazines or newspapers (adjusted OR = 1.6, *P* < .001), billboards (adjusted OR = 1.4, *P* = .002), internet (adjusted OR = 1.8, *P* < .001), television (adjusted OR = 1.4, *P* < .001) and radio (adjusted OR = 1.7, *P* < .001). Exposure to marijuana advertisements from the internet was associated with increased use of marijuana (β = 0.3, *P* = .04).

**Conclusion:**

Exposure to marijuana advertisements was associated with higher odds of current marijuana use among adolescents. Regulations that limit marijuana advertisements to adolescents and educational campaigns on harmfulness of illicit marijuana use are needed.

## Introduction

Marijuana is the most widely used illicit drug by adolescents in the United States; 14.0% of adolescents in grades 8, 10, and 12 reported use of marijuana in the past 30 days in 2015 (1). Marijuana use declined among adolescents from 1979 to 1992 and then rebounded in the 1990s (1). Marijuana use among adolescents has been steady since 2000, but the perceived harmfulness of using marijuana is softening and concurrent use of marijuana and other drugs is rising (1). An estimated 19.8 million people aged 12 years or older used marijuana in the past month and 8.1 million used it on 20 or more days in 2013 (2). Adolescents are in a transition stage, in which marijuana use could cause damage to brain development and lead to concurrent use of stronger substances such as cocaine and opioids (3,4). Adolescents are likely to be influenced by advertisements to accept and normalize substance use behaviors. Tobacco advertising and smoking behaviors are strongly associated (5–7). Similar correlations exist between exposure to advertisements and drinking among adolescents (8,9). Because many states have legalized marijuana use in some form and some states also have laws legalizing marijuana use for both medical and recreational purposes (10), marijuana has grown into a multibillion-dollar business, and sales are expected to reach $22.8 billion by 2020, up from $5.7 billion in 2015 (11). In the last several years, marijuana has received increased attention from the media (12,13), and advertisements for marijuana have increased (14).

A few studies examined the relationship between exposure to marijuana advertisements and marijuana use among adolescents (15–17). D’Amico and colleagues (15) surveyed 6th-, 7th-, and 8th-graders from 16 middle schools in southern California and found that exposure to medical marijuana advertisements was significantly associated with adolescents’ intention to use marijuana and their actual marijuana use 1 year later; the study, however, did not differentiate among channels of exposure (eg, stores, billboards, television, internet). Other studies focused on exposure to television advertisements (16,17). Little is known about how exposure to marijuana advertisements is associated with the prevalence and frequency of marijuana use among adolescents at the national level and whether they differ by various channels of exposures and sociodemographic characteristics (eg, race/ethnicity, age, sex).

To address gaps in knowledge, this study analyzed the exposure to marijuana advertisements through multiple channels by using a nationally representative sample of middle and high school students, from the 2014 and 2015 Monitoring the Future (MTF) studies, and assessed the associations between exposure to marijuana advertisements and prevalence and frequency of marijuana use. This study sought to 1) report the prevalence of adolescents’ exposure to marijuana advertisements from stores, magazines or newspapers, billboards, internet, television, and radio; 2) assess associations between exposure to marijuana advertisements from each channel and marijuana use (current use and frequency of use), and 3) examine whether the number of exposures to marijuana advertisements and other sociodemographic factors are associated with prevalence and frequency of current marijuana use.

## Methods

Data were from the 2014 and 2015 MTF surveys, which are cross-sectional surveys that use a nationally representative sample to explore changes in values, behaviors, and lifestyle orientations among students in the United States. MTF annually surveys 8th-, 10th-, and 12th-grade students by using a multistage, stratified research design and has reported data on drug use for 12th-graders since 1975 and 8th- and 10th-graders since 1991. Details of sampling design are available elsewhere (18). A total of 41,551 students participated in the 2014 survey; response rates were 90% (8th grade), 88% (10th grade), and 82% (12th-grade) (19,20). A total of 44,892 students participated in the 2015 survey; response rates were 89% (8th grade), 87% (10th grade), and 83% (12th grade) (21,22).

In the 2014 and 2015 MFT surveys, 4 randomly distributed forms were used in 8th and 10th grades, and 6 randomly distributed forms were used in 12th grade. National weights were applied to each student record to account for the complex survey design and adjust for nonresponse. Because the data were de-identified and publicly available, the study was considered as non–human subjects research by the institutional review board at Children’s Mercy Hospital.

### Measures

**Marijuana use (current use and frequency of use).** Current marijuana use was defined by the item “On how many occasions (if any) have you used marijuana (grass, pot) or hashish (hash, hash oil) . . . during the last 30 days?” Response options were 1 (0 occasions), 2 (1 or 2 occasions), 3 (3–5 occasions), 4 (6–9 occasions), 5 (10–19 occasions), 6 (20–39 occasions), and 7 (≥40 occasions). Students who reported 0 occasions were defined as not using marijuana. Current marijuana users were defined as students who reported at least a 1-time use of marijuana in the last 30 days. Frequency of marijuana use among current users was measured as a continuous variable (from 2 to 7).

**Exposure to advertisements.** Exposure to marijuana advertisements on storefronts, in magazines or newspapers, on billboards, on the internet, on television, and on radio was measured by the following 6 items in the MTF: 1) “In recent months, about how often have you seen advertisements promoting marijuana use . . . A: on storefronts? B: on billboards? C: in magazines or newspapers? D: on the Internet? E: in TV? F: on the radio?” Response options included: “Not at all,” “Less than once a month,” “1–3 times per month,” “1–3 times per week,” “Daily or almost daily,” and “More than once a day.” For each type of advertisement, students who responded “not at all” were defined as having no exposure to marijuana advertisements, and those who chose any of the other responses were defined as having exposure. The number of exposures from multiple channels was further summed as exposure from 0, 1, 2, 3, and 4 or more types of channels.

Exposure to marijuana advertisements was asked in one form in 8th and 10th grades (form 4) and 2 forms in 12th grade (forms 1 and 2). Of all respondents to the 2014 and 2015 MTF studies combined (n = 86,443), 12,988 (15.0%) were randomly assigned to the questionnaires that included exposure to marijuana advertisement questions and thus were included in the final analysis. These 2 groups (those who got the questionnaires about marijuana advertisements and those who did not) had similar sociodemographic profiles.

**Covariates.** Several covariates were included to assess social factors that influence marijuana use: sex (male and female), race/ethnicity (black, white, and Hispanic), grade (8th, 10th, and 12th grade with the typical age of 13, 15, and 17 years old, respectively), parental education level (highest level of education of either parent, categorized as less than high school diploma, high school diploma, and college or higher), geographic residence region based on census categories (Northeast, North Central, South, and West), and population density (large metropolitan statistical area [MSA], other MSA, and non-MSA).

### Statistical analysis

Weighted percentages of sociodemographic characteristics, marijuana use (current use and frequency use), and exposure to marijuana advertisements by channel were calculated and compared by using the Rao–Scott χ^2^ test. Survey logistic regression models were used to examine the associations between exposure to marijuana advertisements and prevalence of marijuana use among adolescents, and survey linear regression models were used to examine the associations between exposure to marijuana advertisements and frequency of marijuana use. Adjusted odds ratios (ORs) and 95% confidence intervals were calculated for the dichotomous dependent variable and a linear regression coefficient and standard error for the continuous dependent variable. Statistical analyses were performed by using SAS version 9.4 (SAS Institute Inc), and a *P* value < .05 was considered significant.

## Results

Among 12,988 adolescents who responded to the questions about exposure to marijuana advertisements from the combined 2014 and 2015 MTF, 13.8% reported current marijuana use (Table 1). Among current marijuana users, 36.7% used it on 1 or 2 occasions and 14.1% used it on 40 or more occasions in the last 30 days. The prevalence and frequency of use were similar for 2014 and 2015. The prevalence of marijuana use increased significantly by grade: 8th grade, 6.2%; 10th grade, 14.8%; and 12th grade, 20.6%. The frequency of marijuana use also increased significantly by grade: 18.7% of 8th-graders, 20.3% of 10th-graders, and 29.2% of 12th-graders used it on at least 20 occasions in the last 30 days. Boys were significantly more likely than girls to report using marijuana more frequently. For example, 16.7% of boys and 9.1% of girls reported using marijuana on 40 or more occasions in the last 30 days, and white adolescents had a significantly lower prevalence of current marijuana use (12.6%) than did black (14.9%) or Hispanic (14.6%) adolescents. Respondents whose parental education was college or higher or who lived in the North Central census region or in a non-MSA had a lower prevalence of current marijuana use than their counterparts did. In the univariate analysis, no significant differences were found in frequency of marijuana use by race/ethnicity, parental education, geographic region, or population density.

**Table 1:** Summary of Demographic Characteristics and Marijuana Use Among US Middle and High School Students, Monitoring the Future Survey, 2014 and 2015^a^

Characteristic	Weighted %^b^ (Unweighted n = 12,988)	Marijuana Use, Weighted %	*P* Value^c^	No. of Occasions of Marijuana Use in Past 30 Days Among Current Users, Weighted %						
				1 or 2	3–5	6–9	10–19	20–39	≥40	*P* Value^c^
**Total^d^**	—	13.8	—	36.7	19.9	9.5	9.6	10.3	14.1	—
**Year**										
2014	47.5	14.1	.48	37.7	20.0	9.7	8.6	9.6	14.4	.80
2015	52.5	13.6		35.7	19.8	9.3	10.6	10.8	13.7	
**Grade **										
8	32.9	6.2	<.001	42.1	23.2	9.9	6.2	6.7	12.0	.001
10	34.8	14.8		41.5	17.6	9.1	11.4	10.0	10.3	
12	32.3	20.6		31.2	20.7	9.7	9.3	11.6	17.6	
**Sex**										
Male	48.4	14.2	.11	30.4	17.9	11.2	11.0	12.8	16.7	<.001
Female	51.6	13.0		43.9	22.6	7.9	8.3	8.3	9.1	
**Race/ethnicity**										
Black	15.2	14.9	.04	37.0	19.4	10.0	8.5	11.7	13.4	.31
White	63.6	12.6		37.6	18.9	9.6	8.7	10.8	14.5	
Hispanic	21.2	14.6		37.2	25.7	9.2	11.7	7.3	8.9	
**Parental education**										
<High school diploma	8.3	16.2	<.001	29.1	21.8	10.4	14.9	7.8	15.9	.23
High school diploma	33.8	16.1		34.7	20.2	10.2	10.1	11.3	13.4	
College or higher	57.9	11.6		41.2	19.5	9.2	9.0	9.8	11.4	
**Region^e^**										
Northeast	17.7	16.3	<.001	40.7	15.5	8.9	11.2	10.6	13.0	.17
North Central	22.1	12.5		36.0	18.0	11.8	8.7	10.1	15.4	
South	37.8	12.7		35.0	19.0	9.1	9.5	12.0	15.4	
West	22.5	14.9		36.1	26.5	8.6	9.2	7.6	12.0	
**Population density**										
Large MSA	31.3	15.0	.004	39.2	19.5	9.0	10.0	10.2	12.0	.65
Other MSA	48.4	14.0		35.9	19.3	10.0	10.0	10.6	14.1	
Non MSA	20.3	11.6		33.8	22.3	8.9	7.6	9.3	18.0	

Abbreviation: MSA, metropolitan statistical area.

a Data sources: Johnston et al (19–22).

b National weights were applied to each student record to account for the complex survey design and adjust for nonresponse.

c Rao–Scott χ^2^ test was used to determine *P* values for the weighted percentages.

d Number of observations missing from each variable: grade (n = 0), sex (n = 594), race/ethnicity (n = 2,582), parental education (n = 1,194), region (n = 0), and population density (n = 0).

e Northeast: Connecticut, Massachusetts, Maine, New Hampshire, New Jersey, New York, Rhode Island, Pennsylvania, and Vermont. North Central: Iowa, Kansas, Illinois, Indiana, Michigan, Minnesota, Missouri, Nebraska, North Dakota, Ohio, South Dakota, and Wisconsin. South: Alabama, Arkansas, Washington, DC, Delaware, Florida, Georgia, Kentucky, Louisiana, Maryland, Mississippi, North Carolina, Oklahoma, South Carolina, Tennessee, Texas, Virginia, and West Virginia. West: Arkansas, Arizona, California, Colorado, Idaho, Hawaii, Montana, Nevada, New Mexico, Oregon, Utah, Washington, and Wyoming.

Exposure to marijuana advertisements was prevalent among adolescents: 52.8% reported exposure from the internet, 32.1% from television, 24.1% from magazines or newspapers, 19.7% from radio, 19.0% from storefronts, and 16.6% from billboards (Table 2). After adjustment for grade, sex, race/ethnicity, parental education, region, and population density, current use of marijuana among adolescents was significantly associated with exposure to marijuana advertisements on storefronts, magazines or newspapers, billboards, internet, television, and radio. The linear regression showed that exposure to marijuana advertisements from the internet was associated with a 0.3-unit increase in the scale (ranging from 2 to 7) of marijuana use frequency (β = 0.3, *P* = .04); no significant associations were found between frequency of marijuana use and other types of exposure.

**Table 2:** Exposure to Marijuana Advertisements and Its Association With Marijuana Use Among US Middle and High School Students, Monitoring the Future Survey, 2014 and 2015^a^

Type of Advertisement	Unweighted No.	Exposed to Advertisement, Weighted %	Marijuana Use, Weighted %	Prevalence of Marijuana Use^b^, Adjusted OR^c^ (95% CI) [*P* Value]	Frequency of Marijuana Use in Last 30 Days Among Current Users, β^d^ (SE) [*P* Value]
**On storefronts**					
No	10,506	81.0	13.1	1 [Reference]	1 [Reference]
Yes	2,418	19.0	16.7	1.4 (1.2–1.6) [<.001]	0.1 (0.1) [.60]
**Magazine or newspaper**					
No	9,772	75.9	12.0	1 [Reference]	1 [Reference]
Yes	3,093	24.1	19.7	1.6 (1.4–1.9) [<.001]	0.2 (0.1) [.19]
**Billboard**					
No	10,813	83.4	13.3	1 [Reference]	1 [Reference]
Yes	2,089	16.6	16.2	1.4 (1.1–1.6) [.002]	0.1 (0.2) [.62]
**Internet**					
No	6,024	47.2	9.7	1 [Reference]	1 [Reference]
Yes	6,850	52.8	17.6	1.8 (1.5–2.1) [<.001]	0.3^c^ (0.1) [.04]
**Television**					
No	8,740	67.9	12.3	1 [Reference]	1 [Reference]
Yes	4,142	32.1	17.0	1.4 (1.2–1.7) [<.001]	0.2 (0.1) [.18]
**Radio**					
No	10,306	80.3	12.4	1 [Reference]	1 [Reference]
Yes	2,543	19.7	20.0	1.7 (1.5–2.0) [<.001]	0.2 (0.1) [0.09]

Abbreviations: CI, confidence interval; OR, odds ratio; SE, standard error.

a Data sources: Johnston et al (19–22).

b Students who reported no current use of marijuana were the reference group.

c Models were adjusted for grade, sex, race/ethnicity, parental education, region, and population density.

d The regression coefficient stands for a 0.3-unit increase in the scale (2–7) of marijuana use frequency. That is, participants exposed to marijuana advertisements from the internet had an average 0.3-unit increase in the scale of marijuana use frequency, which was coded as a continuous variable with 1 = 0 occasions, 2 = 1 or 2 occasions, 3 = 3–5 occasions, 4 = 6–9 occasions, 5 = 10–19 occasions, 6 = 20–39 occasions, and 7 = 40 or more occasions.

Overall, 58.7% of respondents reported some level of exposure to marijuana advertisements in recent months; 19.8% reported exposure from 1 type of channel, 12.2% from 2 types of channels, 8.4% from 3 types of channels, and 18.3% from 4 or more types of channels. Exposure to marijuana advertisements from multiple channels was associated with increased odds of reporting current marijuana use. Compared with adolescents with no exposure to marijuana advertisements, adolescents who reported exposure from 1 channel had a 60% increase in odds of being current marijuana users (Table 3); exposure from 2 types of channels increased the odds by 70%, and exposure from 3 or more types of channels more than doubled the odds. Tenth-graders had 3.2 times the odds and 12th-graders 4.0 times the odds of 8th-graders of reporting marijuana use. Girls were less likely than boys to report use of marijuana, and black adolescents had higher odds of reporting marijuana use than did white adolescents. Adolescents whose parents had a college education or higher had lower odds of reporting marijuana use than adolescents whose parents did not have a high school diploma. Adolescents who lived in North Central, South, and West census regions were less likely to report marijuana use than those living in the Northeast. Respondents from a large MSA or other MSA were more likely to report marijuana use than those from a non-MSA.

**Table 3:** Associations Between Level of Exposure to Marijuana Advertisements in Recent Months and Current Marijuana Use Among US Middle and High School Students, Monitoring the Future Survey, 2014 and 2015^a^

Characteristic	Prevalence of Marijuana Use^b^ (n = 12,988)		Frequency of Marijuana Use in Last 30 Days Among Current Users (n = 1,806)	
	Adjusted OR^c^ (95% CI)	*P* Value	β^c^ (SE)	*P* Value
**No. of exposure**s				
0	1 [Reference]		1 [Reference]	
1	1.6 (1.3–2.0)	<.001	0.07 (0.2)	.67
2	1.7 (1.4–2.1)	<.001	0.32 (0.2)	.07
3	2.1 (1.6–2.7)	<.001	0.30 (0.2)	.17
≥4	2.1 (1.7–2.6)	<.001	0.27 (0.2)	.11
**Grade**				
8	1 [Reference]		1 [Reference]	
10	3.2 (2.5–3.9)	<.001	0.01 (0.2)	.95
12	4.0 (3.2–5.0)	<.001	0.23 (0.2)	.26
**Sex**				
Male	1 [Reference]		1 [Reference]	
Female	0.9 (0.7–1.0)	.048	−0.70 (0.1)	<.001
**Race/ethnicity**				
White	1 [Reference]		1 [Reference]	
Black	1.3 (1.0–1.6)	.03	0.01 (0.2)	.97
Hispanic	1.0 (0.8–1.2)	.76	−0.31 (0.1)	.04
**Parental education**				
<High school diploma	1 [Reference]		1 [Reference]	
High school diploma	0.9 (0.7–1.2)	.45	−0.32 (0.2)	.09
College or higher	0.6 (0.5–0.8)	.001	−0.53 (0.2)	.006
**Region**				
Northeast	1 [Reference]		1 [Reference]	
North Central	0.7 (0.6–0.9)	.004	0.08 (0.2)	.66
South	0.7 (0.6–0.9)	.004	0.09 (0.2)	.57
West	0.7 (0.6–0.9)	.009	−0.32 (0.2)	.08
**Population density **				
Large MSA	1.4 (1.1–1.7)	.004	−0.23 (0.2)	.20
Other MSA	1.2 (1.0–1.5)	.03	0.002 (0.2)	.99
Non MSA	1 [Reference]		1 [Reference]	

Abbreviations: CI, confidence interval; MSA, metropolitan statistical area; OR, odds ratio; SE, standard error.

a Data sources: Johnston et al (19–22).

b Students who reported no current use of marijuana were the reference group.

c Adjusted ORs and regression coefficients were adjusted for grade, sex, race/ethnicity, parental education, region, and population density in the multivariable analysis.

Exposure to marijuana advertisements from multiple channels was not associated with frequency of marijuana use. Sex, race/ethnicity, and parental education were 3 factors that were associated with frequency of marijuana use in the multivariable analysis. Girls were less likely than boys to report using marijuana more frequently (β = −0.7, *P* < .001). Hispanic adolescents were less likely than white adolescents to report using marijuana more frequently (β = −0.3, *P* = .04). Students whose parents had a college education or more were less likely than those whose parents did not have a high school diploma to report using marijuana more frequently (β = −0.5, *P* = .006).

## Discussion

This study found that exposure to marijuana advertisements is prevalent among adolescents. More than half of MTF respondents in 2014 and 2015 reported some level of exposure to marijuana advertisements. Exposures were through a wide range of media channels, and the internet was the most common channel, followed by television, magazines or newspapers, radio, stores, and billboards, in that order. It is not surprising that adolescents reported the greatest exposure through the internet, because adolescents spend a substantial amount of time online. According to a 2015 Pew Research Center report, 92% of teenagers go online daily, including 24% who say they go online “almost constantly” (23). Digital media, including social media sites, were reported to be common sources for observing marijuana advertising (24). The marijuana industry might follow the similar strategy from tobacco industry (25) to entice adolescents so that they might become regular users in the future. Federal and state regulations on marijuana advertisements are needed to prevention exposure among adolescents. Colorado passed rules to restrict retail marijuana establishments from using television, radio, print, and internet advertisements for adolescents under the age of 21 (26). Surveillance of exposure to marijuana advertisements among adolescents is warranted.

This study showed that exposure to marijuana advertisements on storefronts, magazines or newspapers, billboards, internet, television, and radio were all significantly associated with current marijuana use among adolescents. For example, after adjustment for sociodemographic characteristics, adolescents with exposure to marijuana advertisements on the internet in recent months were 80% more likely to report marijuana use in the last 30 days than adolescents with no such exposure. Furthermore, adolescents usually were exposed to marijuana advertisements through multiple channels, and 38.9% of adolescents reported exposure to marijuana advertisements from at least 2 types of channels. Exposure to marijuana advertisements through multiple channels further increased the odds of current marijuana use. Although this cross-sectional study precludes causal inferences, these results are consistent with the results of a previous study on middle school adolescents’ marijuana use in southern California (15). This study also confirmed that exposure to marijuana advertisements might have similar effects on adolescents’ marijuana use that exposures to advertisements for other substances (eg, alcohol, cigarettes, e-cigarettes) have on adolescents’ use of those substances. Moreover, exposure to marijuana advertisements from the internet was also associated with reporting marijuana use more frequently in the last 30 days. Exposure to marijuana advertisements could influence how adolescents perceive the drug and normalize their use of it, resulting in an increase in the use of marijuana. As more states approve recreational marijuana use or pass medical marijuana provisions (27), researchers should assess the impact of advertisements on adolescents’ marijuana use and inform policy initiatives for regulating marijuana advertisements. People who work closely with adolescents, such as parents, pediatricians, clinicians, and school educators, should educate adolescents about the harmfulness of marijuana use, such as poor school performance and neuropsychological performance deficits (28), to counter the effects from exposure to marijuana advertisements (15).

This study identified other sociodemographic factors associated with current marijuana use and frequency of marijuana use. Older (vs younger) adolescents, boys (vs girls), black adolescents (vs white adolescents), adolescents whose parents did not have a high school diploma (vs college or higher), those who lived in Northeast (vs other regions) or in a large or other MSA (vs non-MSA) were more likely to be current marijuana users. Sex, race/ethnicity, and parental education level were also associated with using marijuana more frequently. For example, Hispanic adolescents were less likely than white adolescents and boys were more likely than girls to use marijuana more frequently. Knowledge of the social factors that contribute to adolescents’ marijuana use can help school administrators and public health professionals develop targeted prevention strategies to prevent adolescents from drug abuse. For example, anti-marijuana use messages tailored to adolescents with certain sociodemographic characteristics might resonate better with students than a one-size-fits-all approach.

This study has several limitations. First, the 2014 and 2015 MTF studies are cross-sectional; thus, this study could not determine whether the exposure to marijuana advertisements caused an increased use of marijuana or vice versa. Second, exposure to advertisements was self-reported. Thus, recall and attentional biases might have existed in this study, especially for younger respondents (29). For example, current marijuana users might be more likely to pay attention to marijuana advertisements than those who are not interested in marijuana (30). Longitudinal studies to evaluate the effects of exposure to marijuana advertisements on marijuana use among adolescents are needed. Third, because the students were asked only whether they had seen advertisements promoting marijuana use, this study did not differentiate between medical and recreational advertisements. The influence of these 2 types of advertisements on adolescents’ marijuana use might differ; future research should evaluate the effects of medical and recreational advertisements separately. Fourth, single-item measures were used to examine exposure to marijuana advertisements, and they might be less psychometrically robust than multiple-item measures. Nevertheless, the strength of this study, with single-item measures, was to allow assessment of the effects of multiple exposures of marijuana advertisements through multiple channels. Fifth, social media sites were reported to be common sources of exposure to marijuana advertisements (24). It is not clear how exposure to social media sites confounded with exposure to the internet in this study; continued surveillance of marijuana advertisements is warranted. Finally, because the MTF study excluded high school dropouts and home-schooled students, who may have a high prevalence of marijuana use (31), this study might have had a sampling bias.

Despite these limitations, this study found that exposure to marijuana advertisements is prevalent among 8th-grade, 10th-grade, and 12-grade adolescents in the United States: 58.7% of MTF respondents reported some level of exposure to marijuana advertisements in recent months. Exposure to marijuana advertisements was significantly associated with higher odds of marijuana use among adolescents. Regulations on marijuana advertisements and educational campaigns on harmfulness of illicit marijuana use are needed.

## References

[1] Johnston LD , O’Malley PM , Miech RA , Bachman JG , Schulenberg JE . Monitoring the Future national survey results on drug use, 1975–2015: overview, key findings on adolescent drug use. Ann Arbor (MI): Institute for Social Research, The University of Michigan; 2016.

[2] Substance Abuse and Mental Health Services Administration. Results from the 2013 National Survey on Drug Use and Health: summary of national findings. Rockville (MD): Substance Abuse and Mental Health Services Administration; 2014.

[3] Lindsay JA , Stotts AL , Green CE , Herin DV , Schmitz JM . Cocaine dependence and concurrent marijuana use: a comparison of clinical characteristics. Am J Drug Alcohol Abuse 2009;35(3):193–8. 10.1080/00952990902933860 19462304 PMC3788597

[4] Subramaniam GA , Ives ML , Stitzer ML , Dennis ML . The added risk of opioid problem use among treatment-seeking youth with marijuana and/or alcohol problem use. Addiction 2010;105(4):686–98. 10.1111/j.1360-0443.2009.02829.x 20403020 PMC2858346

[5] Dai H , Hao J . Exposure to advertisements and susceptibility to electronic cigarette use among youth. J Adolesc Health 2016;59(6):620–6. 10.1016/j.jadohealth.2016.06.013 27528472

[6] Duke JC , Allen JA , Eggers ME , Nonnemaker J , Farrelly MC . Exploring differences in youth perceptions of the effectiveness of electronic cigarette television advertisements. Nicotine Tob Res 2016;18(5):1382–6. 10.1093/ntr/ntv264 26706908

[7] Pucci LG , Siegel M . Exposure to brand-specific cigarette advertising in magazines and its impact on youth smoking. Prev Med 1999;29(5):313–20. 10.1006/pmed.1999.0554 10564621

[8] Anderson P , de Bruijn A , Angus K , Gordon R , Hastings G . Impact of alcohol advertising and media exposure on adolescent alcohol use: a systematic review of longitudinal studies. Alcohol 2009;44(3):229–43. 10.1093/alcalc/agn115 19144976

[9] Snyder LB , Milici FF , Slater M , Sun H , Strizhakova Y . Effects of alcohol advertising exposure on drinking among youth. Arch Pediatr Adolesc Med 2006;160(1):18–24. 10.1001/archpedi.160.1.18 16389206

[10] Governing data. State marijuana laws map. 2016. http://www.governing.com/gov-data/state-marijuana-laws-map-medical-recreational.html. Accessed April 19, 2016.

[11] Hughes T . Legal marijuana sales forecast to hit $23B in 4 years. USA Today. March 20, 2016. http://www.usatoday.com/story/money/business/2016/03/20/legal-marijuana-sales-forecast-hit-23b-4-years/82046018/.

[12] Brosious E . Marijuana shops now outnumber McDonald’s and Starbucks in Oregon. Suntimes. November 8, 2015. http://www.businessinsider.com/there-are-more-marijuana-shops-in-oregon-than-starbucks-and-mcdonalds-2015-.

[13] CBS News. “60 Minutes” examines effects of marijuana on body. Aired on October 28, 2016. http://www.cbsnews.com/videos/60-minutes-examines-effects-of-marijuana-on-body/.

[14] Schroyer J . Industry snapshot: marketing & advertising. Marijuana Business Magazine. https://mjbizmagazine.com/industry-snapshot-marketing-advertising/. Accessed July 13, 2017.

[15] D’Amico EJ , Miles JN , Tucker JS . Gateway to curiosity: medical marijuana ads and intention and use during middle school. Psychol Addict Behav 2015;29(3):613–9. 10.1037/adb0000094 26030167 PMC4587352

[16] Alvaro EM , Crano WD , Siegel JT , Hohman Z , Johnson I , Nakawaki B . Adolescents’ attitudes toward antimarijuana ads, usage intentions, and actual marijuana usage. Psychol Addict Behav 2013;27(4):1027–35. 10.1037/a0031960 23528197 PMC4480868

[17] Huansuriya T , Siegel JT , Crano WD . Parent–child drug communication: pathway from parents’ ad exposure to youth’s marijuana use intention. J Health Commun 2014;19(2):244–59. 10.1080/10810730.2013.811326 24308793

[18] Bachman J , Johnston L , O’Malley P , Schulenberg J . The Monitoring the Future project after thirty-seven years: design and procedures. Occasional paper no. 76, 2011. http://www.monitoringthefuture.org/pubs/occpapers/mtf-occ76.pdf. Accessed on June 7, 2017.

[19] Johnston LD , Bachman JG , O’Malley PM , Schulenberg JE , Miech RA . National Addiction & HIV Data Archive Program. Monitoring the Future: a continuing study of American youth (12th-grade survey), 2014 (ICPSR 36263). Ann Arbor (MI): Institute for Social Research, Survey Research Center. http://www.icpsr.umich.edu/icpsrweb/NAHDAP/studies/36263/version/2. Accessed October 24, 2017.

[20] Johnston LD , Bachman JG , O’Malley PM , Schulenberg JE , Miech RA . National Addiction & HIV Data Archive Program. Monitoring the Future: a continuing study of American youth (8th- and 10th-grade surveys), 2014 (ICPSR 36149). Ann Arbor (MI): Institute for Social Research, Survey Research Center. http://www.icpsr.umich.edu/icpsrweb/NAHDAP/studies/36149/version/1. Accessed October 24, 2017.

[21] Johnston LD , Bachman JG , O’Malley PM , Schulenberg JE , Miech RA . National Addiction & HIV Data Archive Program. Monitoring the Future: a continuing study of American youth (12th-grade survey), 2015 (ICPSR 36408). Ann Arbor (MI): Institute for Social Research, Survey Research Center. http://www.icpsr.umich.edu/icpsrweb/NAHDAP/studies/36408/version/1. Accessed October 24, 2017.

[22] Johnston LD , Bachman JG , O’Malley PM , Schulenberg JE , Miech RA . National Addiction & HIV Data Archive Program. Monitoring the Future: a continuing study of American youth (8th- and 10th-grade surveys), 2015 (ICPSR 36407). Ann Arbor (MI): Institute for Social Research, Survey Research Center. http://www.icpsr.umich.edu/icpsrweb/NAHDAP/studies/36407/version/1. Accessed October 24, 2017.

[23] Lenhart A . Teens, social media & technology overview 2015. Pew Research Center. http://www.pewinternet.org/2015/04/09/teens-social-media-technology-2015/#teens. Accessed June 12, 2016.

[24] Krauss MJ , Sowles SJ , Sehi A , Spitznagel EL , Berg CJ , Bierut LJ , Marijuana advertising exposure among current marijuana users in the U.S. Drug Alcohol Depend 2017;174:192–200. 10.1016/j.drugalcdep.2017.01.017 28365173 PMC5436304

[25] US Department of Health and Human Services. Preventing tobacco use among youth and young adults: a report of the Surgeon General. Atlanta (GA): US Department of Health and Human Services, Centers for Disease Control and Prevention, National Center for Chronic Disease Prevention and Health Promotion, Office on Smoking and Health; 2012.

[26] Colorado Department of Revenue. Permanent rules related to the Colorado retail marijuana code. 2013. https://www.colorado.gov/pacific/sites/default/files/Retail%20Marijuana%20Rules,%20Adopted%20090913,%20Effective%20101513%5B1%5D_0.pdf. Accessed August 1, 2017.

[27] Ingraham C . Marijuana wins big on election night. Washington Post. November 8, 2016. https://www.washingtonpost.com/news/wonk/wp/2016/11/08/medical-marijuana-sails-to-victory-in-florida/. Accessed October 24, 2017.

[28] Hall W . The adverse health effects of cannabis use: what are they, and what are their implications for policy? Int J Drug Policy 2009;20(6):458–66. 10.1016/j.drugpo.2009.02.013 19362460

[29] Brener ND , Billy JO , Grady WR . Assessment of factors affecting the validity of self-reported health-risk behavior among adolescents: evidence from the scientific literature. J Adolesc Health 2003;33(6):436–57. 10.1016/S1054-139X(03)00052-1 14642706

[30] Hanewinkel R , Isensee B , Sargent JD , Morgenstern M . Cigarette advertising and teen smoking initiation. Pediatrics 2011;127(2):e271–8. 10.1542/peds.2010-2934 21242217

[31] Townsend L , Flisher AJ , King G . A systematic review of the relationship between high school dropout and substance use. Clin Child Fam Psychol Rev 2007;10(4):295–317. 10.1007/s10567-007-0023-7 17636403

